# Usability Testing of a Digital Assessment Routing Tool: Protocol for an Iterative Convergent Mixed Methods Study

**DOI:** 10.2196/27205

**Published:** 2021-05-18

**Authors:** Cabella Lowe, Harry Hanuman Sing, Mitchell Browne, Meshari F Alwashmi, William Marsh, Dylan Morrissey

**Affiliations:** 1 Centre for Sports & Exercise Medicine William Harvey Research Institute Queen Mary University of London London United Kingdom; 2 Faculty of Medicine Memorial University St John’s, NL Canada; 3 Risk and Information Systems Research Group School of Electronic Engineering and Computer Science Queen Mary University of London London United Kingdom; 4 Physiotherapy Department Barts Health NHS Trust London United Kingdom

**Keywords:** mHealth, mobile health, eHealth, digital health, digital technology, musculoskeletal injury, musculoskeletal conditions, triage, physiotherapy triage, usability, acceptability

## Abstract

**Background:**

Musculoskeletal conditions account for 16% of global disability, resulting in a negative effect on millions of patients and an increasing burden on health care utilization. Digital technologies that improve health care outcomes and efficiency are considered a priority; however, innovations are often inadequately developed and poorly adopted. Further, they are rarely tested with sufficient rigor in clinical trials—the gold standard for clinical proof of efficacy. We have developed a new musculoskeletal Digital Assessment Routing Tool (DART) that allows users to self-assess and be directed to the right care. DART requires usability testing in preparation for clinical trials.

**Objective:**

This study will use the iterative convergent mixed methods design to assess and mitigate all serious usability issues to optimize user experience and adoption. Using this methodology, we will provide justifiable confidence to progress to full-scale randomized controlled trials when DART is integrated into clinical management pathways. This study protocol will provide a blueprint for future usability studies of mobile health solutions.

**Methods:**

We will collect qualitative and quantitative data from 20-30 participants aged 18 years and older for 4 months. The exact number of participants recruited will be dependent on the number of iterative cycles required to reach the study end points. Building on previous internal testing and stakeholder involvement, quantitative data collection is defined by the constructs within the ISO 9241-210-2019 standard and the system usability scale, providing a usability score for DART. Guided by the participant responses to quantitative questioning, the researcher will focus the qualitative data collection on specific usability problems. These will then be graded to provide the rationale for further DART system improvements throughout the iterative cycles.

**Results:**

This study received approval from the Queen Mary University of London Ethics of Research Committee (QMREC2018/48/048) on June 4, 2020. At manuscript submission, study recruitment was on-going, with data collection to be completed and results published in 2021.

**Conclusions:**

This study will provide evidence concerning mobile health DART system usability and acceptance determining system improvements required to support user adoption and minimize suboptimal system usability as a potential confounder within subsequent noninferiority clinical trials. Success should produce a safe effective system with excellent usability, facilitating quicker and easier patient access to appropriate care while reducing the burden on primary and secondary care musculoskeletal services. This deliberately rigorous approach to mobile health innovation could be used as a guide for other developers of similar apps.

**International Registered Report Identifier (IRRID):**

DERR1-10.2196/27205

## Introduction

### Background

Musculoskeletal conditions are recognized as a global issue, with between 20%-33% of people living with a painful musculoskeletal condition. These conditions are the highest contributor to global disability at 16%, resulting in a negative effect on millions of patients and an increasing burden on health care utilization [1]. Musculoskeletal conditions are prevalent throughout the lifespan and are associated with early work retirement and reduced ability to participate socially [2]. In developed countries, they present the most significant proportion of lost productivity in the workplace, leading to a significant impact on the gross domestic product and health care costs [3-6]. Musculoskeletal conditions can affect as many as 1 in 4 adults and are set to continue rising, being associated with increased life expectancy and reduced activity [4,5]. Access to the “right person, right place, first time” is considered a key factor in improving musculoskeletal condition outcomes and in reducing unwarranted variations in clinical pathways, such as unnecessary secondary care consultations and investigations [7]. Musculoskeletal triage as a single point of access is effective across various outcome measures, including user satisfaction, diagnostic agreement, appropriateness of referral, and reduction in patient waiting times [8]. Importantly, triage has also shown a reduction in cost across the musculoskeletal pathway, which is particularly crucial in overburdened health care systems, where triage can be performed effectively via several methods and by a range of clinicians [9-11]. For example, the National Health Service England is introducing physiotherapists as musculoskeletal first-contact practitioners; however, this is dependent on the recruitment of a significant number of clinicians with the associated challenges [7]. Mobile health (mHealth) technology is proposed as a cost-effective solution for improving health care delivery [12,13]. Although many mHealth tools have not demonstrated cost-effectiveness or have shown merely to shift spending to another part of the health system [14], it would seem logical that a digital alternative to physio-led triage, able to replicate the same stratification of care and reduction in costs, is a desirable objective. Thus, Optima Health has developed the Digital Assessment Routing Tool (DART) mHealth system to assess the patient’s musculoskeletal presentation through a series of questions and responses accessed via their mobile devices. The patient is taken through a subjective assessment, which is driven by 9 sets of clinical algorithms that cover all body regions, similar to a clinician performing a virtual triage by telephone or videocall. At the end of the assessment, the patient is given a signposting recommendation to one of the clinical services available to them within the musculoskeletal pathway. The objective is to provide patients with easily accessible, safe, and effective access to musculoskeletal services, while releasing valuable clinical resources to consistently work at the top of their skill set, where they add the greatest value to the patient journey. The development of more remote health care delivery options has been greatly accelerated by the COVID-19 pandemic and the benefits offered by a virtual approach are likely to support further uptake of systems such as DART. Patients are already making their own decisions about accessing health care for a range of conditions or increasingly turning to unvalidated self-help apps for advice that can be unhelpful or even potentially harmful [15,16]. Guidance for safe and effective development of mHealth apps has been published by several national and international organizations [17-20]. While these recommendations provide advice for developers, it is recognized that there remains no single regulatory framework to which all mHealth developments must conform [21] and that work remains to develop a suitable legal framework [22].

**Figure 1:**
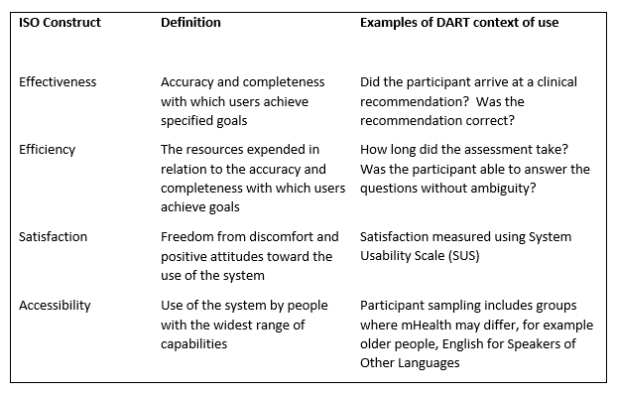
ISO 9241-210:2019 constructs and definitions and examples of digital assessment routing tool context of use. DART: Digital Assessment Routing Tool; mHealth: mobile health.

Up to 2017, there was an exponential increase in the rate of mHealth app releases, resulting in over 259,000 apps available in just the major app stores alone [23,24]. However, many mHealth apps have subsequently fallen, with many unsuccessful attempts to scale up from a prototype to successful implementation. Inattention to usability during the design and testing phases has been cited as the potential cause [25-27], contributing to the high abandonment rate [28]. Usability is crucial in the development of mHealth systems and is reflected within guidance documents relating to standards for design, development, testing, and implementation of these devices [17-19]. Usability has been defined as the extent to which a system can be used by specified users to achieve specified goals with effectiveness, efficiency, and satisfaction in a specified context of use [19]. These constructs will all be assessed during the study (Figure 1). However, there is little agreement about the most effective methodology for usability testing, with researchers using a combination of different design components to assess mHealth apps [29]. The iterative convergent mixed methods design used for this research will provide a more holistic assessment of DART compared with other mHealth studies. Although quantitative questionnaires are the most frequently used method for assessing mHealth system usability [29], they are unlikely to identify the specific problems that need to be addressed. Questionnaire scores only give a general overview of usability, without providing the level of understanding of usability problems required for system iteration. Qualitative studies alone are not able to provide a definitive level of acceptability of a system. Iteration is recognized as a key enabler of successful products but is not included by most developers, where the usability testing of a final version is often immediately prior to deployment [29]. The inclusion of patient and public involvement during mHealth testing allows users to bring their own personal perspectives into the process, giving researchers the understanding of usability issues that would not have otherwise been recognized [30]. This study will combine all these elements into a methodology specifically developed to assess an mHealth triage or symptom checker. The iterative approach of cyclical evaluation and improvement plus mixed methods allows richness while quantifying use, thus maximizing usability and system adoption.

### DART Overview

**Figure 2:**
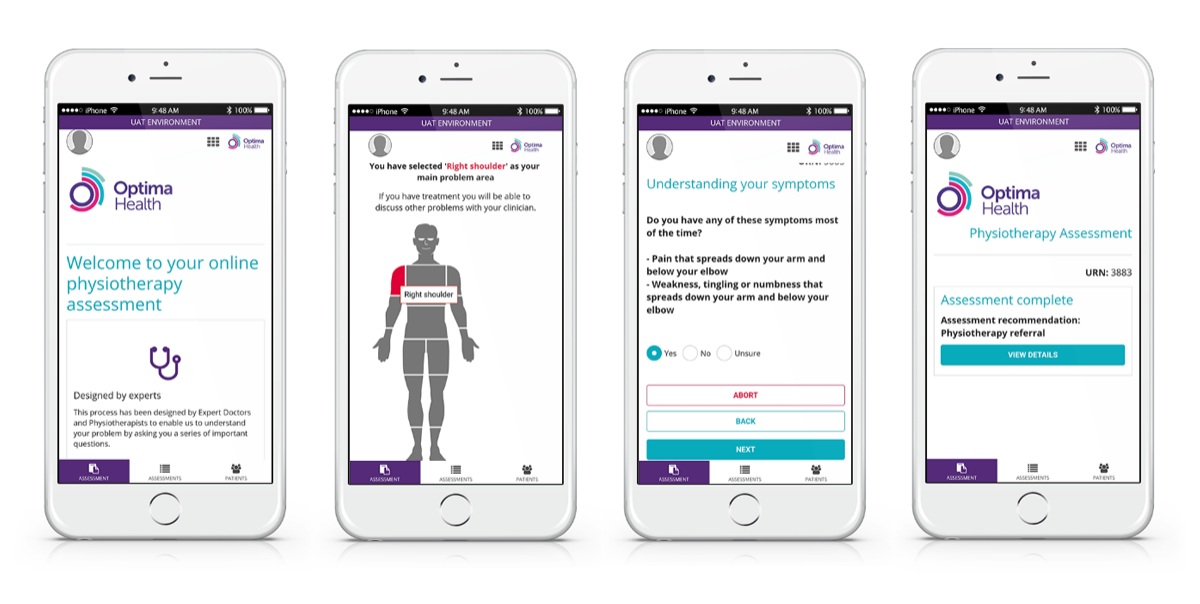
Digital Assessment Routing Tool mobile health system.

DART is a first-contact mHealth system utilizing 9 musculoskeletal clinical algorithms, configured to provide the patient with a recommendation to the correct intervention level (Figure 2). Designed specifically for managing musculoskeletal conditions, it delivers a narrower but deeper assessment than that found with more generic symptom checkers. The patient can self-assess using a computer, tablet, or smartphone. Alternatively, the content can be delivered by a remotely situated clinician or a nonclinical administrator by telephone or videocall. The patient selects the body region related to his/her primary problem and is then presented with a varying number of questions, depending on the nature of his/her symptoms and previous responses. Serious pathology is identified and signposted at the start of the assessment, with less urgent medical referrals being identified as the patient passes down the questioning. Algorithms are configured to match the provider’s clinical services based on evidence-based practice and sector-specific referral criteria. DART can be applied across any number of health care systems, including public and private services. DART typically signposts to emergency or routine medical assessment, specific condition specialists, physiotherapy, self-management programs, or psychological support services. Referral thresholds can be configured to match service requirements, such as increasing the volume of patients directed to self-management or physiotherapy. Situated next to the questions are information boxes, which assist the patient in answering the question, thereby improving the accuracy of the responses. DART has an integral reporting function, thereby allowing the analysis of individual and amalgamated patient data to assess the system and clinical pathway performance.

### Previous Work

This usability study is part of a larger project, bringing DART from concept to implementation through a series of clinical and academic research work packages. To assess algorithm clinical validity, 2 reports were commissioned by Optima Health and undertaken by a panel of 5 consultant clinicians prominent in the musculoskeletal field. The first round of desktop evaluation consisted of experts inputting symptoms from 98 clinical scenarios (including red flags and complex presentations) into DART. The DART recommendation was assessed by the expert as being correct, arguably correct, or disagree. Feedback from the experts was incorporated into a new iteration, leading to improved DART accuracy during the second panel review. Based on their opinions, the panel recommended that the clinical validity was sufficient to allow DART to proceed to further research studies. The initial usability study protocol went through a series of iterations within an internal review process, comprising the research project team and DART system developers to arrive at the final version presented in this paper. Using a new usability testing methodology, this study will provide a rich understanding of how users interact with DART and guide further iterations. The impact of this will be to optimize usability before evaluating the safety and effectiveness of DART in a randomized controlled trial.

### Research Aim, Objectives, and End Points

The aim of this study is to assess and optimize DART usability, which could result in maximizing user adoption. The objectives are as follows: (1) to understand what users consider to be strengths and weakness of using DART, (2) to identify usability issues and map to a usability problem grade to inform the next DART iteration development, and (3) to complete a cycle of iterations until usability reaches a predefined acceptable level. The end points are as follows: (1) all Grade 1 and 2 usability problems have been mitigated following a minimum of 3 user group sessions and (2) system usability scale score is 80 or greater after a minimum of 3 user group sessions plus 1 additional session, representing a “good” or better system.

## Methods

### Study Design

**Figure 3:**
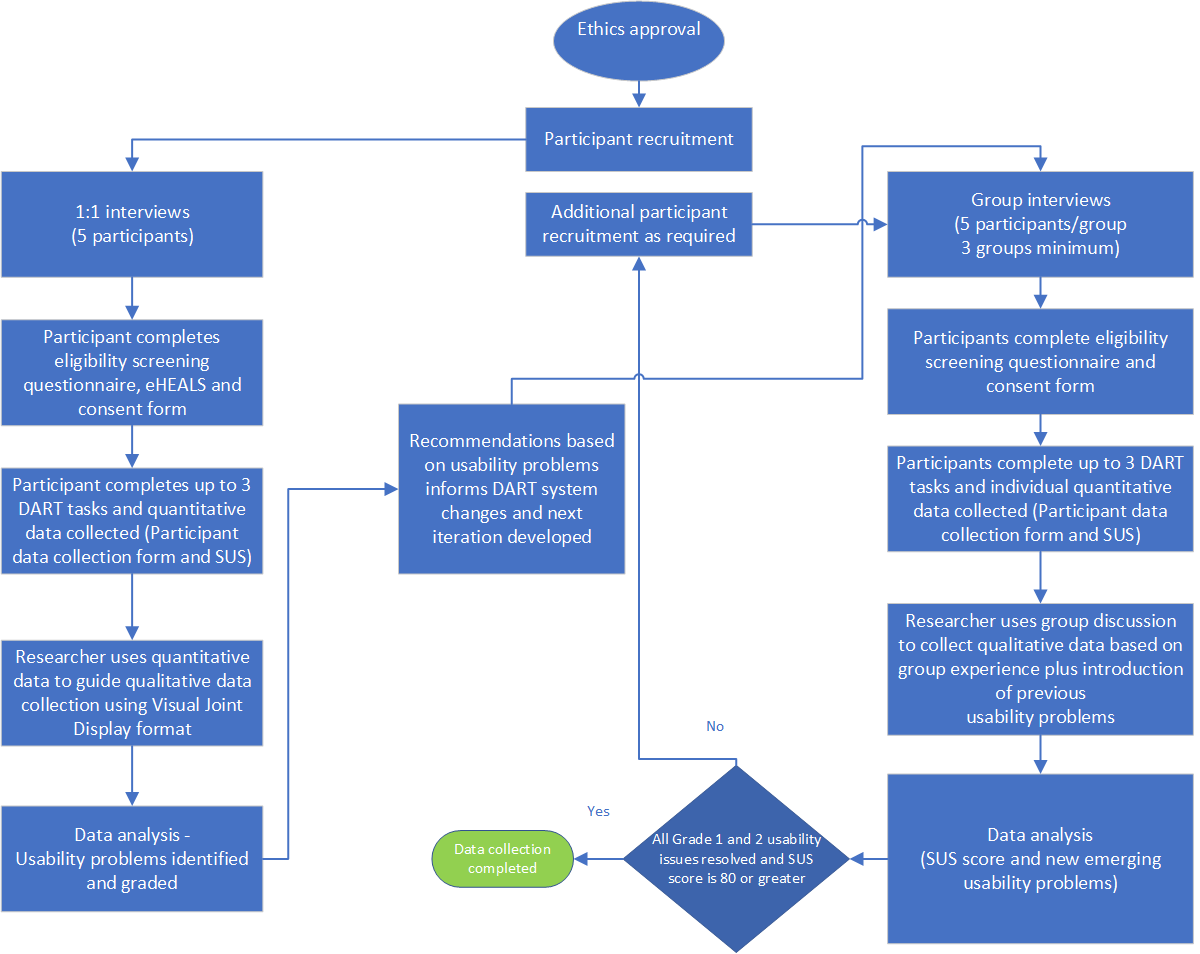
Digital Assessment Routing Tool usability study iterative convergent mixed methods design. DART: Digital Assessment Routing Tool; SUS: system usability scale.

This study will use an iterative convergent mixed methods design, as described by Alwashmi et al [31]. The collection and integration of quantitative and qualitative data of direct relevance to the DART mHealth app will be used to inform subsequent DART usability improvements (Figure 3). The first phase of data collection will consist of 5 interviews with individual participants who have used the tool to identify key usability issues and gain a baseline system usability scale score. It has been suggested that 5 participants are likely to expose about 80% of the usability issues [32]. Although it is recognized that the DART target population will be heterogenous, the first round of interviews is expected to expose key issues from the sample. This will be followed by group sessions to capture a greater diversity of potential DART user population to improve validity of the data. Owing to logistic requirements, interview sessions will be conducted remotely using Microsoft Teams videoconferencing software and web-based questionnaires.

### Participant Recruitment

A stratified purposive sampling method will be used to gather information from participants able to access the internet as well as to explore the scope of potential DART accessibility [33]. We will use a criterion-based selection, categorizing participant characteristics of age, internet use, sex, English for Speakers of Other Languages groups—all of which are subgroups that have shown to contribute small differences in internet use [34]. To ensure that participants are included from groups potentially less likely or able to use mHealth systems, a sampling matrix will be used, which provides quotas specifying the number of people required for each characteristic [33]. Recruitment of 20-30 participants will be via convenience sampling and snowballing; study recruitment will continue until there is a representative sample from each category defined by the sample matrix and the study end points being reached. Recruitment material will be distributed to local community groups, Optima Health’s existing client base of employers and their staff, plus Queen Mary University of London (QMUL) students. Potential participants expressing an interest will be sent a patient information sheet and consent form (Multimedia Appendix 1). They will have an opportunity to review this material and if they wish to proceed, they will be registered for the study.

### Inclusion Criteria

The study participant inclusion criteria are as follows: (1) adults older than 18 years; (2) able to speak and read English; (3) live in the United Kingdom; (4) access the internet at least once every 3 months; (5) has access to a smartphone, tablet, or laptop; and (6) current or previous experience of a musculoskeletal condition.

### Exclusion Criteria

The study participant exclusion criteria are as follows: (1) significant visual or memory impairment sufficient to affect the ability to answer questions and recall information in an individual or group discussion setting; (2) medically trained, musculoskeletal health care professional, for example, doctor, physiotherapist; (3) relatives or friends of the researchers; and (4) Optima Health employees.

### Study Duration

It is anticipated that this study will last for up to 3 months after receiving the ethical approval. However, this will be dependent on the number of DART iterations required to achieve the end points. Participants who have raised high-grade usability problems will be invited to participate in a subsequent study session to assess the impact of DART system changes.

### Theoretical Framework

**Figure 4:**
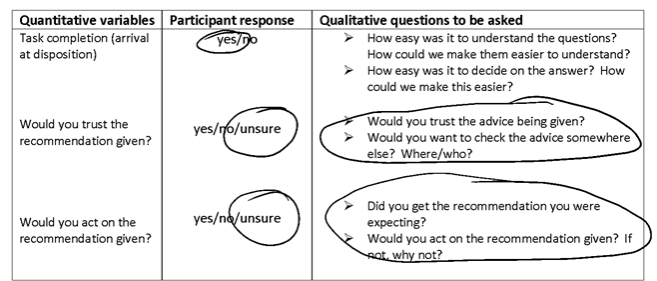
An extract from a joint visual display showing how a researcher uses responses to quantitative data to guide qualitative data collection in real time.

The iterative convergent mixed methods design, as described by Alwashmi [31], involves simultaneous qualitative and quantitative data collection and analysis that continues cyclically through rounds of mixed methods data collection and analysis until the mHealth technology under evaluation is found to work to the agreed criteria. This design will be used to enhance the usability of the DART system by using strategies of matching, diffracting, and expanding. The *matching* process involves aligning qualitative questions with quantitative variables which, in this study, are defined by the constructs within the ISO 9241-210-2019 standard [19] and the system usability scale [35-37]. These are presented as a visual joint display [38], which allows the researcher to explore usability themes as they emerge during the data collection. As the participant responds to the quantitative questioning, the researcher highlights in real time areas on the visual joint display that they wish to explore in more detail during the qualitative element of data capture (Figure 4). *Diffraction* allows the addition of qualitative questions that explore different aspects of the quantitative data that are not addressed through the scales or items being collected. Examples for this study include trust and intention to act, as well as the importance of clinical escalation. *Expanding* further supports the ability of the researcher to examine findings from the divergence of the qualitative and quantitative data, exploring different aspects of a single phenomenon. This could include an instance where the DART task completion time may be longer for some participants, to whom spending longer understanding and answering the questions accurately is more important than being able to finish the assessment quickly.

### Data Collection

Following consent being given, participants will complete a short questionnaire providing demographic data. Five one-to-one sessions scheduled to last up to 60 minutes will be conducted using Microsoft Teams (videoconferencing facility developed by Microsoft) video calls. After completing the eHEALS health literacy questionnaire [39], the participant will be asked to log in to DART. Using an existing or previous musculoskeletal condition to complete the assessment, they will be encouraged to give feedback using the concurrent think-aloud method [40]. This can be repeated for up to 3 different conditions. They will then complete 2 quantitative data questionnaires concerning ISO effectiveness, efficiency, satisfaction, and accessibility constructs. Satisfaction will be measured using the system usability scale. This data will inform and direct subsequent qualitative data collection. The researcher will identify variables with low scores, for example, “I found the product unnecessarily complex” and focus their qualitative questions to target these areas, interactively considering both types of data in the context of each other. The interviews will be recorded and transcribed verbatim using Otter (automated video and audio transcription software developed by otter.ai), with a final review for accuracy by the researcher using the original recording for comparison. As the study progresses and usability issues are addressed during each iteration, the emphasis for data collection moves from being largely qualitative to become more focused on the quantitative data driving the study end points. Up to 5 participants will attend a web-based session where they will be asked to explore DART. They will complete the quantitative data questionnaires and be invited to discuss any comments they may have. Qualitative questioning at this stage will be broader in nature, with postuse debrief questions as follows: (1) were you asked about what was important to you? (2) how did you find the questions? and (3) could we improve the system?

The researcher will use this opportunity to raise any previous usability problems to assess the impact of changes made to the previous iteration. The group sessions will be recorded and transcribed.

### Data Analysis

**Figure 5:**
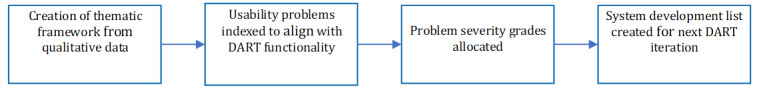
Qualitative data translates into system improvement in the iterative convergent mixed methods design. DART: Digital Assessment Routing Tool.

Qualitative data will be transcribed and analyzed using thematic analysis by 2 researchers working independently using NVivo (qualitative data analysis software developed by QSR International) to create an initial thematic framework (Figure 5). Data will be indexed into usability problems of key importance to the study and presented in a data summary display. The researchers working initially independently and then together will achieve a consensus and agree on allocating a problem severity grade to each data theme. This is obtained by considering the impact of the problem on the user and the frequency with which it occurs, which leads to a decision on the risk of not addressing the problem versus the reward of correcting it. Once the problems have been allocated a severity grade, matched recommendations are given for each to guide system development of the next DART iteration. Actions to address all Grade 1 and 2 usability problems are completed for the next iteration, with Grade 3 and 4 problems remaining on record and reassessed and, if necessary, regraded after each round of testing. After each round of testing, participant system usability scale raw scores will be converted and reported, together with a cumulated score across rounds of testing. The final mean score will also be converted into a percentile score to allow benchmarking against other web-based systems [41]. Participant responses to the following questions were also analyzed and reported: (1) did you get a recommendation? (2) would you trust the recommendation you were given? and (3) would you act on the given recommendation? If the participant is willing to discuss the details of their musculoskeletal conditions, the researcher (a physiotherapist) will provide an opinion regarding whether they thought the DART signposting was arguably correct. Usability will also be considered in the context of participant eHealth literacy.

### Bias

This study is funded by the developers of DART, Optima Health and therefore is at risk of bias. The lead researcher is an employee of Optima Health and enrolled in a PhD program at QMUL. The second researcher, with no connections to Optima Health, will participate in data collection and analysis. To mitigate bias, participants are excluded if they are employees of Optima Health or QMUL or if they are relatives or friends of the lead researcher. Participants will not have seen or used DART previously. To address unconscious bias during the recruitment process, the use of a purposive sampling framework will ensure that people fulfilling the criteria of central importance to the research objectives are included in the study. The 2 study researchers will work independently for the data collection and initial analysis before combining their results and coming to a consensus for usability problem grading. Quantitative data will be collected by the researcher who has no connection to Optima Health or DART system development.

### Risks and Benefits

There is no form of physical intervention during this study and participants are interviewed remotely, with no travel being required. A possible benefit, albeit unlikely, is that a previously undiagnosed serious pathology could be revealed during the testing process and the researcher (a physiotherapist) could immediately advise the participant on the most appropriate action to ensure their safety. Following completion of the DART assessment by the participant, the researcher will discuss the DART disposition with the participant in the context of his/her symptom presentation. If indicated during this discussion, the researcher will also provide appropriate clinical advice and reassurance about the condition management to the participant.

### Informed Consent

Each participant will receive the participant information sheet and consent form (Multimedia Appendix 1), which outlines the purpose of the study and the nature of the participation. This includes information about the format of the interaction (one-to-one or group), potential risks, confidentiality and protection of personal data, the anonymity of study findings, and the right to withdraw at any time without prejudice. After reading the participant information sheet, the participant is given the opportunity to email or request a call with the lead researcher to discuss any questions. The participant will be required to provide written, signed consent prior to any data collection, which will then be posted or emailed to the lead researcher.

### Data Management

Participants have the right to withdraw from the study at any time. If they do, data collected up to the point they withdraw will be retained, but not then added to. Research data will be stored separately to personal data and linked by a unique reference number only accessible to the researchers. Electronic and paper data will be managed and stored securely in accordance with general data protection regulations.

## Results

Ethics approval was received from QMUL Ethics of Research Committee (QMREC2018/48/048) on June 4, 2020. At manuscript submission, the first round of individual interviews has been completed and recruitment commenced for the group sessions. Results will be reported in a follow-up paper later in 2021.

## Discussion

### Overview of This Study

A systematic evaluation of the DART mHealth system in line with the international and national guidelines [17-20] will provide a more precise assessment of its usability and potential adoption. It will also address areas of poor usability that could otherwise become confounding factors within the subsequent noninferiority trial, where DART will be compared with current practice virtual physiotherapy triage. To date, there are no published studies evaluating similar musculoskeletal mHealth systems or indeed the knowledge of clinician error rates in usual care against which to benchmark DART. For this reason, we have set the target usability standard of 80 to be achieved, which will place DART in the top 10% of products tested using the system usability scale [41]. Usability testing will rely on an iterative convergent mixed methods design, which allows data collection specific to the functionality of the DART system, while incorporating validated and widely published usability quality measures. The iterative approach ensures that system changes made in response to identified usability problems are retested by study participants to validate the success of the updates. Based on the data collected, this section will benchmark DART usability as an app, discuss the limitations of the evaluation, and consider essential implications for future DART testing and deployment. The study methodology has been designed to rigorously test the DART mHealth app and could be adopted by other researchers to improve usability and adoption of other similar systems.

### Methodological Limitations

The purpose of this study is to assess usability problems that could influence DART signposting, routing, and user acceptance. This study will not determine the safety and effectiveness of DART, as this will be the subject of a subsequent study. Some limitations have already been identified, such as the challenge of recruiting a representative section of a potentially large and diverse DART user population. The study design is proportionate to the resources available to deliver the study, and it is acknowledged that a more significant number of participants could yield a richer data set. Owing to the limitation of the study resources and the current iteration of DART only being available in the English language, participants unable to read English are excluded. Moreover, because of logistic constraints, all interviews and group sessions will be conducted remotely by video call. It is not known how this may impact the richness or quality of data collection, although this has been considered and attempts will be made to mitigate possible negative effects, which includes ensuring all participants are briefed in advance on how to use the key features of Microsoft Teams and are supported with this during data collection. It is recognized that within virtual group sessions, the researcher will be required to take a more proactive approach to running the session, putting people at ease, and inviting everyone in turn to share their views.

### Methodological Strengths

It is rare that this level of evaluation of an mHealth system is completed [29], with none published in the musculoskeletal field [42]. This protocol provides a template for other researchers and developers to use across triage and referral mHealth systems. The protocol was research in and of itself and has the unique benefit of combining widely accepted methods of assessing system usability together with important factors specific to clinical practice, making it generalizable across systems and adaptable for specific clinical pathways. The incorporation of stakeholder engagement during the study design ensures links between the protocol and the published mHealth system design standards, including requirements for transparency of the research. This protocol is suitable for remote delivery, supporting efficient cost-effective research within the constraints of the social distancing required by the current pandemic restrictions.

## Appendix

Multimedia Appendix 1 Patient information and consent form.

## Abbreviations

DART: Digital Assessment Routing Tool
mHealth: mobile health
QMUL: Queen Mary University of London
