# Supplementary material for: Usability Testing of a Digital Assessment Routing Tool: Protocol for an Iterative Convergent Mixed Methods Study
Source: JMIR Res Protoc. 2021 May 18;10(5):e27205. doi: 10.2196/27205 (PMC8170557; doi:10.2196/27205)
Supplement: Multimedia Appendix 1 [file resprot_v10i5e27205_app1.doc]

# Participant Information Sheet

Research study: “Usability testing of a Digital Assessment Routing Tool: An Iterative Convergent Mixed Methods Study”.

Queen Mary Ethics of Research Committee Ref: **QMREC2018/48/048**

We would like to invite you to be part of this research project, if you would like to. You should only agree to take part if you want to; it is entirely up to you. If you choose not to take part, there won’t be any disadvantages for you and you will hear no more about it.

Please read the following information carefully before you decide to take part. This will tell you why the research is being done and what you will be asked to do if you take part. Please ask if there is anything that is not clear or if you would like more information. You are still free to withdraw at any time and without giving a reason.

**Why are we doing this study?**

One in five people in the UK suffer from a musculoskeletal condition (a problem with their joints, muscles or other soft tissues). There are treatments that can help, including physiotherapy, but sometimes it can be difficult to get to see the right person quickly. Some people need to see their GP first, or may be asked to speak to a physiotherapist on the phone. We are looking at a new way for people to use their computer or mobile device to complete an assessment that will signpost them to the best help for their problem. This will hopefully allow people to get the right help for their condition sooner, which will allow them to recover quicker.

**How can you help us?**

We are contacting you as you have told us that you have (or have had) a musculoskeletal problem and may be interested in taking part in the study. As part of testing the new system, which is called DART (Digital Assessment Routing Tool), we need to understand how people would use it and if there any improvements we could make. We will be conducting a series of interviews to get people’s views and ideas. Our aim is to make these improvements to DART before we test it with real-life patients.

**What will the study involve?**

If you decide to take part in the study, you will asked to complete two on-line forms, which should take about 10 minutes to complete. These will provide us with more information including your name, gender, age, if English is your first language and how you use the internet.

For the main part of the study, you will be invited to attend either a one-to-one interview with the researcher, or a group session with up to four other volunteers and the researcher. We will use an on-line conferencing system for the interviews, which has video and sound. The sessions will be recorded and transcribed into words for the study team to analyze. The sessions will last about an hour in total and the atmosphere will be relaxed. You will be given some background information about DART and then told how to logon to the system using your own mobile device. We will ask you to think about up to three musculoskeletal problems, ones you have now, or have had in the past and use DART to complete an assessment of the problem. You can complete up to three separate assessments for three separate problems. Following this, the researcher will ask you to complete two on-line questionnaires. The researcher will then ask you some questions about what you thought of the system. There are no right or wrong answers. The researcher finally asks if you are happy to discuss more details about the problems you used for the testing, which will help them to understand the accuracy of the system. You do not have to share these details if you would rather not. You do not need to prepare for the session in advance.

**How will the information I give be analysed?**

The study team will analyse the results of the on-line questionnaires, information from the DART on how you used the system and read through the transcript of your session several times. They will note any ideas and phrases, then compare these to other participants. As more people give their ideas, we will be able to see themes start to emerge. When the study team are satisfied that there are no new topics coming from the sessions, they will stop collecting new information. The main themes will be discussed in more detail, using quotations from the interviews as examples. All quotations will be anonymous, meaning that nobody reading the results will be able to tell who was interviewed. The full transcript of your contribution to the session will be available should you want it.

**Ethics and consent**

This study has been given ethical approval by the Queen Mary, University of London (QMUL) Research and Ethics Committee. If you would like to participate in the study, you will need to read and sign the consent form we have sent you with this information sheet and email or post it back to us. Prior to your session, we will check that you are familiar with the information on this sheet and answer any questions you may have. We will also check that you are still happy to proceed with the session. This confirms you are satisfied with how the study has been explained and that you agree to take part. You will also be asked to complete two short questionnaires before the interview starts. This covers some personal information including your name, age, gender, ethnicity and how you use the internet. We will ask you if you would like to share more information about the injuries you thought of when you were testing DART, but you do not have to if you do not want to.

**Maintaining confidentiality**

All electronic information, including your signed consent form and questionnaires, will be stored on password-protected Queen Mary University of London computers and only accessed by the study team. Personal information will be stored separately to the session data. For participants to remain anonymous, each will be identified by a unique code rather than by their name. It is up to the principal investigator to maintain the security of data.

**What if I decide to withdraw?**

You have the right to withdraw at any time during the study without giving a reason why. If you do, your data will be destroyed in a secure manner in accordance with the UK Policy Framework for Health and Social Care Research, however any analysis already completed will not be changed.

**Are there any risks of taking part?**

We do not think that there will be any increased physical risks associated with you taking part in this study as it will be conducted on-line and can be done from home. We will not ask you to discuss any medical information other than the injuries you thought of to test DART. You may want to share your experience of seeking help for a musculoskeletal condition, but this is up to you. You should not find the session stressful, but if you do, you should mention it to the study team. If you suspect a breach of data protection or confidentiality, you should discuss this with the study team.

**Funding**

We will not offer any payment for your participation in this study.

**Will I find out about the results?**

Study findings will be presented as part of the research element of Annual QMUL Scientific Conference.

This study will form part of a larger doctoral research project developing DART and we hope that this will be published in a peer-reviewed medical or scientific journal. You are welcome to ask the study team for the results of the study if you wish.

**Final information**

It is up to you to decide whether to take part. If you do decide to take part, you will be given this information sheet to keep and be asked to sign a Consent Form.

Please read Queen Mary’s privacy notice for research participants[[1]](#footnote-2) for important information about your personal data and your rights in this respect. If you have any questions relating to data protection, please contact Data Protection Officer, Queens’ Building, Mile End Road, London, E1 4NS or [data-protection@qmul.ac.uk](mailto:data-protection@qmul.ac.uk)

If you have any questions or concerns about the way the study is conducted please, in the first instance, contact the researcher responsible for the study. Their details are. Cabella Lowe, email: [c.lowe@qmul.ac.uk](mailto:c.lowe@qmul.ac.uk). Tel: 07976 315105

If this is unsuccessful, or not appropriate, please contact the Facilitator of the Queen Mary Ethics of Research Committee:

Address: Room W104, Queen’s Building, Mile End Campus, Mile End Road, London, E1 4NS. E-mail: [research-ethics@qmul.ac.uk](mailto:research-ethics@qmul.ac.uk)

**Consent Form**

Please complete this form after you have read the Information Sheet and/or listened to an explanation about the research.

Research study: “Usability testing of a Digital Assessment Routing Tool: An Iterative Convergent Mixed Methods Study”.

Queen Mary Ethics of Research Committee Ref: **QMREC2018/48/048**

Thank you for considering taking part in this research. The person organising the research must explain the project to you before you agree to take part.

If you have any questions arising from the Information Sheet or explanation already given to you, please ask the researcher before you decide whether to join in. You will be given a copy of the Information Sheet and this Consent Form to keep and refer to at any time. If you are willing to participate in this study, please initial the appropriate statements and sign and date the declaration underneath.

| **Statement** | **Please initial your agreement with each statement** |
| --- | --- |
| I agree that the research project named above has been explained to me to my satisfaction in verbal and/or written form |  |
| I understand that if I decide at any other time during the research that I no longer wish to participate in this project, I can notify the researchers involved and be withdrawn from it immediately |  |
| I have read both the notes written above and the Information Sheet about the project, and understand what the research study involves |  |
| I agree to take part in the study, which will include use of my data |  |
| I understand that such information will be treated as strictly confidential and handled in accordance with the provisions of the Data Protection Act 1998. |  |
| I understand that the video/audio sessions will be recorded to allow data analysis. |  |

**Participant’s Signature:**  Date:

**Investigator’s Signature:**

I ___________________________________________ confirm that I have carefully explained the nature, demands and any foreseeable risks (where applicable) of the proposed research to the participant and provided a copy of this form.

**Signature:** Date:

1. This is found at: <http://www.arcs.qmul.ac.uk/media/arcs/policyzone/Privacy-Notice-for-Research-Participants.pdf> [↑](#footnote-ref-2)
